# The Effect of Maternal Helminth Infection on Maternal and Neonatal Immune Function and Immunity to Tuberculosis

**DOI:** 10.1371/journal.pone.0093429

**Published:** 2014-04-07

**Authors:** Dawit Gebreegziabiher, Kassu Desta, Girmay Desalegn, Rawleigh Howe, Markos Abebe

**Affiliations:** 1 Armauer Hansen Research Institute (AHRI), Addis Ababa, Ethiopia; 2 Department of Medical Laboratory Sciences, Addis Ababa University, Addis Ababa, Ethiopia; 3 Mekelle University, Mekele, Ethiopia; Fundació Institut d'Investigació en Ciències de la Salut Germans Trias i Pujol. Universitat Autònoma de Barcelona. CIBERES, Spain

## Abstract

**Background:**

*M. tuberculosis* and helminth infection each affects one third of the world population. Helminth infections down regulate cell mediated immune responses and this may contribute to lower efficacy of BCG vaccination and higher prevalence of tuberculosis.

**Objective:**

To determine the effect of maternal helminth infection on maternal and neonatal immune function and immunity to TB.

**Methods:**

In this cross sectional study, eighty five pregnant women were screened for parasitic and latent TB infections using Kato-Katz and QFT-GIT tests, respectively. IFN-γ and IL-4 ELISpot on Cord blood Mononuclear Cells, and total IgE and TB specific IgG ELISA on cord blood plasma was performed to investigate the possible effect of maternal helminth and/or latent TB co-infection on maternal and neonatal immune function and immunity to TB.

**Result:**

The prevalence of helminth infections in pregnant women was 27% (n = 23), with *Schistosoma mansoni* the most common helminth species observed (20% of women were infected). Among the total of 85 study participants 25.8% were QFT-GIT positive and 17% had an indeterminate result. The mean total IgE value of cord blood was significantly higher in helminth positive than negative women (0.76 vs 0.47, p = 0.042). Cross placental transfer of TB specific IgG was significantly higher in helminth positive (21.9±7.9) than negative (12.3±5.1), *p* = 0.002) Latent TB Infection positive participants. The IFN-γ response of CBMCs to ESAT-6/CFP-10 cocktail (50 vs 116, *p* = 0.018) and PPD (58 vs 123, *p* = 0.02) was significantly lower in helminth positive than negative participants. There was no significant difference in IL-4 response of CBMCs between helminth negative and positive participants.

**Conclusions:**

Maternal helminth infection had a significant association with the IFN-γ response of CBMCs, total IgE and cross placental transfer of TB specific IgG. Therefore, further studies should be conducted to determine the effect of these factors on neonatal immune response to BCG vaccination.

## Introduction

Over one third of the human population is currently infected with *M. tuberculosis,* and a similar percentage with helminths, the majority of these infections are found in the developing countries [1]–[3]. About 90–95% of the *M. tuberculosis* infected individuals develops latent tuberculosis infections (LTBI). Active tuberculosis (TB) results either from uncontrolled primary infection or reactivation of LTBI, particularly in young children, pregnant mothers and immunocompromised individual [4]–[6]. Between 8 and 9 million people develop active TB each year, and about two million die from TB every year [7], [8]. Though rare, there is also risk of transmission from mother to child [9]–[11]. The major immune response during *M. tuberculosis* infection (both active and latent) is cell mediated. Studies suggested that CD4 T cells (primarily T_H_1) play a critical role, the effector function of which is mainly mediated by the production of IFN-γ [12]. The role of humoral immunity is also documented in the literature as *M. tuberculosis* specific antibodies significantly enhance complement fixation and complement mediated phagocytosis [13]. However, the host immune response to *M. tuberculosis* infection is often impaired due to other co-infections such as helminths [14]. Studies conducted in developing countries indicated that women of childbearing age are frequently infected with one or more helminths [15],[16]. This may be due to immunological and physiological changes during pregnancy which favors persistent infection [17], [18]. If left untreated, their infections will persist throughout the period of pregnancy or longer, and produce millions of eggs per day, accompanied by copious amounts of secretary and excretory helminth products crossing the placenta, potentially influencing the fetal immune system. *In utero* stimulation with helminth-derived antigens is believed to divert fetal immunity towards T_H_2 responses and/or lead to anergy or tolerance [19]–[22].

Although helminths have extensive species diversity, in the majority of cases the immune responses are remarkably similar, dominated by IL-4, IL-5, IL-10 and TGF-β cytokines; and leading to strong IgE, eosinophil, and mast cell responses [23]–[28]. Such immune responses attenuate T_H_1 type cytokine production which may increase susceptibility of the host to TB [27], [29]–[33]. There is also increasing evidence that prenatal T-cell priming of the fetal immune system can occur via trans-placental exposure to the helminth derived antigens and such primary immune sensitization can affect the appropriate maturation of the postnatal immune responses [21], [34], [35]. *In utero* exposure to helminth derived antigens has been considered as one of the risk factors in offspring for enhanced susceptibility to infections such as TB [19], [25], [36]. Therefore, we hypothesized that maternal helminth infection significantly affects Cord Blood Mononuclear Cells (CBMCs) T_H_1/T_H_2 cytokine responses and increases total IgE concentration, and the transplacental accumulation of maternal TB specific IgG in cord blood plasma [37].

## Materials and Methods

A cross-sectional design was used to determine the effect of maternal helminth infections on maternal and neonatal immune function and immunity to TB. This study was conducted in Mekelle, the Tigray regional state, in Northern Ethiopia from October 2011 to July 2012 after obtaining institutional approval from Addis Ababa University, Armauer Hansen Research Institute and Mekelle University. Consecutive samples were collected from 85 voluntary pregnant women at the last week of their ninth month of pregnancy from MCH (Maternal and Child Health) departments of Mekelle Hospital, Semen Health Center and Ayder Referral Hospital. Only HIV negative pregnant mothers were included in the study.

### Parasitological examination

Stool samples were collected in screw capped containers and transported within 30 minutes to Tigray Regional Laboratory for processing. Duplicate Kato slides were prepared within one hour of collection and examined within two days of collection. Wet mount preparation was also done to evaluate for hook worm and protozoan infections within 30 minutes. Following delivery, all women who were positive for any species of parasite were treated accordingly.

### Diagnosis of LTBI using QuantiFERON-TB Gold In-Tube Assay

All study participants were screened for LTBI using QFT-GIT (Cellestis, Limited, Carnegie, Victoria, Australia). One milliliter of blood was collected into each of the three tubes; the Nil (negative control), the mitogen (positive control), and the *MTB* antigens (ESAT-6, CFP-10, and TB7.7) and incubated at 37°C for 20 hours. Plasma was harvested by centrifuging at 3000 rpm for 15 minutes, and stored at −80°C until transported to the AHRI laboratory using dry ice. Plasma was thawed and ELISA was performed according to the QFT-GIT kit and the optical density (OD) was measured using a microplate ELISA reader (Molecular Devices Corporation, USA) fitted with a 450 nm and 650 nm filters. The actual IFN-γ concentration in the samples was calculated based on curves generated from standards using QuantiFERON-TB Gold Analysis Software supplied by Cellestis, Limited, Carnegie, Victoria, Australia.

The assay result was positive if the net IFN-γ response to the TB antigens was ≥0.35 IU/ml, regardless of the mitogen response, and negative if the net IFN-γ response was <0.35 IU/ml with net mitogen response (>0.5 IU/ml). A result is said to be indeterminate if there was excessive IFN-γ production from the negative control (>8.0 IU/ml) or insufficient net IFN-γ response from mitogen (<0.5 IU/ml) and net TB antigen response (<0.35 IU/ml).

### Plasma and CBMC isolation

Cord blood was collected from 85 full-term uncomplicated pregnancies with heparinized tubes by trained midwives. To avoid mixing with maternal blood, cord blood was collected by directly inserting a needle fitted with 20 ml syringe to the vein of umbilical cord. Plasma was separated by centrifugation at 1500 rpm, 10 min and stored at −80°C until transported to AHRI laboratory. The cord blood was 1∶1 diluted with complete media (RPMI containing 10% fetal calf serum (FCS), 1% Penicillin and Streptomycin (P/S) and 1% L-glutamine) and CBMCs were isolated by Ficoll-Hypaque density gradient technique, washed three times with complete media and kept frozen in freezing medium (10% dimethyl sulfoxide (DMSO) in FCS) at −80°C.

### Thawing of cord blood mononuclear cells and preparation for ELISPOT

Cryopreserved CBMCs were thawed in a 37°C water bath. Cell suspensions were transferred into a 15 ml blue cap. Complete media was added slowly and stepwise up to 12 ml and washed 3 times by centrifuging the tubes for 10 minutes at 1600 rpm. After discarding the supernatant, the pellet was re-suspended in 1 ml complete media. Cell number and cell viability was determined using trypan blue and the number of live CBMCs was adjusted to 2×10^5^ cells/well in complete media.

### Total IgE ELISA assay

Plasma was thawed and ELISA was performed using human total IgE ELISA kits (product code 3810-1H-6, Mabtech, Sweden). Microwell plates were coated with 2 µg/ml anti-total IgE monoclonal antibody (mAb 107) and incubated overnight at 4°C. After plates were blocked by adding 200 µl/well of 0.05% PBS Tween 20 containing 0.1% BSA and incubated for 1 hour at room temperature, 100 µl/well of plasma and the serially diluted standards were added to wells based on the plate layout. Biotinylated monoclonal detection antibody was added and after 1 hour incubation, a 1∶1000 dilution of streptavidin-HRP was added and incubated for 1 hour. At each step, wells were washed five times using PBS containing 0.05% Tween 20. O-phenylenediaminedihydrochloride (OPD) tablet was used as substrate for color development. ODs were measured at 450 nm on a multiwell plate ELISA reader. GraphPad PRISM statistical software, inc, USA was used to convert the ODs to total IgE concentration values.

### TB specific IgG ELISA assay

A TB specific IgG ELISA was performed using the Diagnostic Automation *Mycobacterium tuberculosis* IgG antibody test kit (Diagnostic Automation, INC, Catalog number 5111-8, USA). Sample plasma diluted1∶101 or ready-to-use standards were pipetted into the wells of the microtiter strips coated with *M. tuberculosis* antigens. After 1 hour incubation, the ready-to-use anti-human-IgG peroxidase conjugate was added and incubated for 30 minutes. Following washing, the substrate (TMB) solution was pipetted and incubated for 20 minutes. The color development was stopped by adding stop solution and measured spectrophotometrically at the wavelength of 450 nm.

### ELISPOT cytokines assays

The frequency of IFN-γ and IL-4 secreting CBMCs were measured using an ELISpot assay in response to ESAT-6/CFP-10 cocktail, PPD (Statens Serum institute, SSI, Denmark) and anti-CD3 stimulation using IFN-γ and IL-4 ELISpot kits (Mabtech, Sweden). PVDF-plates (Millipore, USA)) were briefly treated with 70% ethanol and coated with anti-IFN-γ monoclonal capture antibody (1-D1K for IFN-γ). Precoated plates were used for IL-4 ELISpot. The final concentration of the antigens was 5 µg/ml. 2×10^5^cells/well in complete medium were added to pre-coated wells based on plate layout. After 24 hours of incubation (37°C, 5% CO_2_), 100 µl/well of biotinylated detection monoclonal antibody was added. Following 1 hour incubation at room temperature, a 1∶1000 diluted streptavidin-ALP was added. Finally, 100 µl/well of filtered substrate (BCIP/NBT-plus) was added. After 30 minutes of incubation at room temperature and vigorous washing using tap water, spots were counted using an ELISpot reader (AID GmBH, Strasburg, Germany) and reported as number of spot forming cells (SFC) per million CBMCs.

### Statistical Analysis

Data was entered and analyzed using the SPSS statistical package version 19. Descriptive statistical analysis was performed for socio-demographic and clinical characteristics. GraphPad PRISM was used to convert ODs of total IgE to actual concentration. Univariate logistic regression was used to explore the association between of QFT-GIT indeterminate result with different clinical data. Multivariate analysis was performed to test the association between demographic risk factors (age, occupation, educational background and number of pregnancies) and parasitic infections. One way ANOVA was used to determine mean difference of total IgE in QFT-GIT positive, negative and indeterminate results. Chi square test was also used to compare QFT-GIT indeterminate results with helminth positive and negatives. Independent sample t-tests were used to statistically evaluate the differences in cord blood plasma total IgE between helminth positive and negative. Since the frequencies of cytokine secreting CBMCs were not normally distributed, nonparametric statistical analysis was used. Kruskal-Wallis and Mann-Whitney tests were used to compare frequencies of IFN-γ and IL-4 CBMCs between the four different groups. The Spearman Rank Order Correlation coefficient was also used to determine the relationship between the frequencies of IFN-γ and IL-4 secreting CBMCs and total IgE values. Analysis of immune response was performed after study participants were grouped based on the status of helminth and latent TB infections. Accordingly, there were four groups: helminth positive-LTBI positive (n = 9), helminth positive-LTBI negative (n = 14), helminth negative-LTBI positive (n = 13) and helminth negative-LTBI negative (n = 35).

### Ethical Consideration

Ethical approval was obtained from the department of Medical laboratory sciences (MLS), College of Allied Health Sciences (CAHS), Addis Ababa University (AAU), and Armauer Hansen Research Institute (AHRI) research ethics review committees. Participants were enrolled in the study after they had been informed about the purpose of the study and signed the written informed consent.

## Results

### Demographic and Clinical Information

The median age of the study participants was 25 years (IQR 22, 28). The educational background of the study participants was: primary school 43(50.6%), secondary school 24(28.2%), tertiary school 9(10.6%) and no formal education 9(10.6%). All of the study participants were urban residents. The majority (83.3%) of study participants were house wives and 11.8% were government employees.

Forty (47%) of the study participants were in their first pregnancy and 25(29.4%) in their second pregnancy. Of the total participants, only 13(15.3%) reported a history of BCG vaccination of which a BCG scar was found only in 20(23.1%) of the participants. Only five of the study participants reported close contact with a TB patient and all of these exposures occurred 5 to 10 years previously.

### Parasitic Infections

The prevalence of parasitic infections was 27.1% (n = 23) and 8.2% (n = 7) for helminths and protozoans respectively. The most prevalent parasite was *Schistosoma mansoni* (20.0%, n = 17) followed by *Ascaris lumboricoid* (8.2%, n = 7), *Entrobius vermicularis* (5.8%, n = 5), *Trichuris trichiura* (4.7%, n = 4), *Hymenolepis nana (2.3%, n = 2), Entamoeba species (8.2%, n = 7) and Giardia lamblia (2.4%, n = 2)*. None of the study participants were found to be positive for *Schistosomia haematobium.* The overall intestinal parasite prevalence was 28.2%. Infection with more than one helminth was observed in 15.3% of the study participants. Helminth-protozoan double infection was observed in 7.1% of the study participants.

The association between demographic risk factors (age, occupation, educational background and number of pregnancies) and parasitic infections was tested using multivariate analysis and a significant association was observed only for educational level and parasitic infection. As the level of education increases from no formal education to primary and then to secondary level, the odds of parasitic infection was decreased (OR = 0.50, CI =  0.211 to 0.992, *p* = 0.048).

### Latent Tuberculosis Infection

The result of QFT-GIT test indicated that LTBI was found in 22(26.8%) while 14(17%) had an indeterminate result. Three of the indeterminate samples were randomly selected and re-tested for reproducibility of the test and they remained indeterminate. All indeterminate results were due to an insufficient IFN-γ response to mitogen. The proportion of QFT-GIT indeterminate results were significantly higher in helminth positive participants than helminth negatives (*p* = 0.048) but this effect was not observed in protozoan infection (*p* = 0.273).

### Total White Cell Count (WBC) and Differential Count in Cord Blood

The overall median value of the cord blood WBC was 13440/mm^3^ (IQR = 11575, 16178). Further analysis showed no statistically significant difference in the median value of cord blood WBC count between helminth positive and negative study participants (*p = 0.714*). Similarly the proportion of lymphocyte, monocyte and granulocyte within the cord blood was not affected by maternal helminth infection (data not shown).

### Total IgE in Cord Blood

The mean value of total IgE of cord blood was significantly lower in helminth negative (0.47±0.43) than helminth positive subjects (0.76±0.59, *p = 0.042, t-test*). Though the aggregate effect of maternal helminth infection was reached at significant level of *p*<0.05, each helminth species had no statistically significant difference.

### TB specific IgG

**Figure 1:**
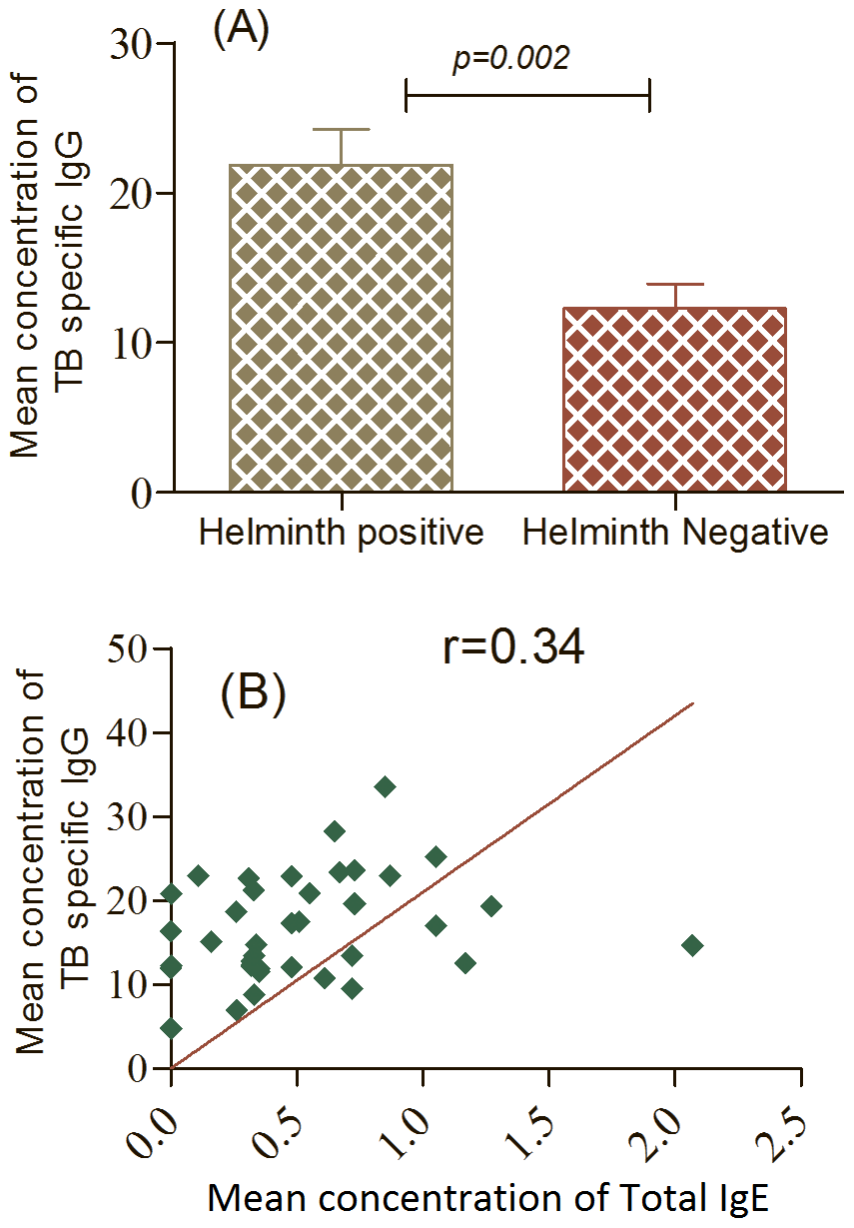
Mean concentration difference of cord blood plasma of TB specific IgG between helminth positive (n = 9) and negative LTBI positives (n = 13)(A) and the correlation of total IgE (n = 80) and TB specific IgG (n = 80)(B). A line through the origin is fitted to indicate the deviation of points from the perfect (r = 1) correlation line.

TB specific IgG ELISAs were performed to evaluate if maternal helminth infections and elevated cord blood IgE had an association with the cross placental transfer of TB specific IgG in 82 of the study participants irrespective of their QFT results. The results showed that 6(7.3%) of the cord blood samples contained TB specific IgG above the cut-off value (*Mycobacterium tuberculosis* IgG, Diagnosis Automation, Inc, catalog number 5111-8, USA). As shown in Figure 1A, the mean concentration value of TB specific IgG in cord blood was significantly lower in helminth negative (12.3±5.1) than positive study participants (21.9±7.9); *p* = 0.002). We further evaluated the correlation between total IgE and TB specific IgG of the cord blood plasma (Figure 1B). The result showed that TB specific IgG and total IgE of cord blood had medium positive statistical significant correlation (r = 0.34, *p* = 0.034) (Fig. 1B).

### ELISpot Assay

*In vitro* IFN-γ and IL-4 secretion were measured using an ELISpot assay after CBMCs were stimulated with TB antigens and results were reported as spot forming cells (SFC)/million cells. Samples with cytokine responses to anti-CD3 positive control below the response to *Mycobacterium* antigens were excluded from the analysis. The CBMCs were compared for frequency of IFN-γ and IL-4 cytokine responses among 22 study participants i.e., [helminth and LTBI co-infected subjects (H^+^L^+^, n = 9), and helminth negative but LTBI positive subjects (H^−^L^+^n = 13)]. The median frequency of IFN-γ spot forming CBMCs was 43 (IQR = 19 to 119), 58 (IQR = 29 to 123) for H^+^L^+^ and 120(IQR = 54 to 231) and 123(IQR = 67 to 265) for H^−^L^+^ in response to ESAT-6/CFP-10 cocktail and PPD in *vitro* stimulation respectively (Table 1).

**Table 1:** The median frequency of IFN-γ and IL-4 secreting CBMCs (n = 22) among H^+^L^+^ (n = 9), and H^−^L^+^ (n = 13).

Groups	Median frequency IFN-γ secreting cells/million			Median frequency of IL-4 secreting cells/million		
	ESAT-6/CFP-10	PPD	Anti-CD_3_	ESAT-6/CFP-10	PPD	Anti-CD_3_
H^+^L^+^	43	58	254	13	19	134
H^−^L^+^	120	123	210	8	14	102

*H^+^L^+^ = helminth & LTBI positive and H^−^L^+^ = helminth negative & LTBI positive.*

**Figure 2:**
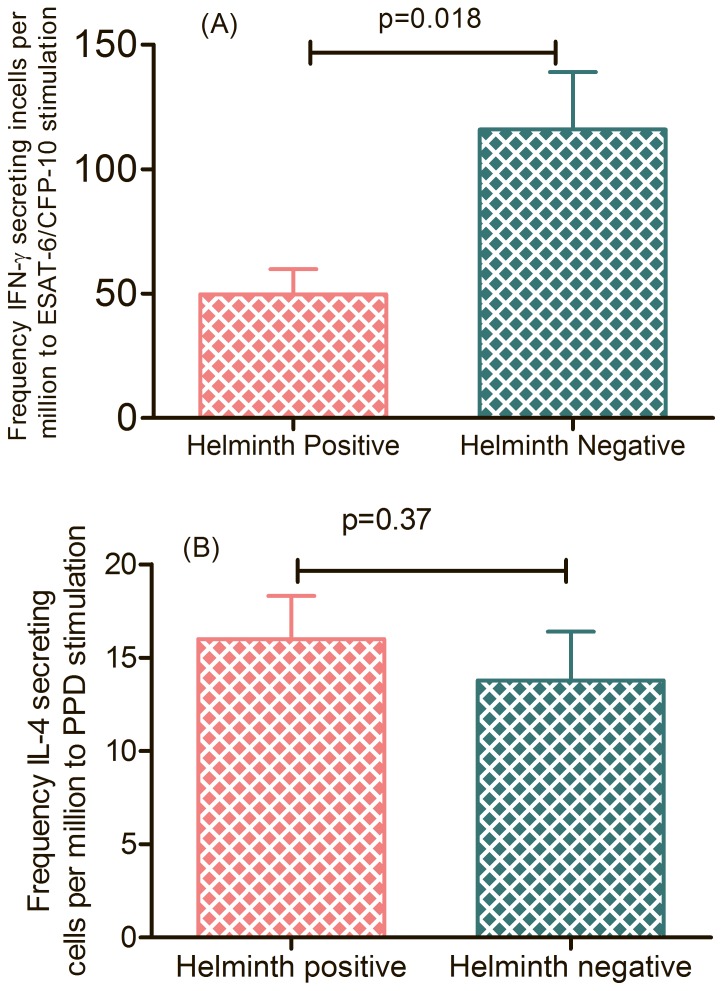
The frequency of IFN-γ (A) and IL-4 (B) secreting CBMCs (n = 40) in response to EAST-6/CFP-10 cocktail stimulation between helminth positive (n = 20) and negative (n = 20) study participants respectively.

After the effect of LTBI and LTB-helminth co-infection was evaluated, study participants were categorized based on helminth infection and the median frequency of IFN-γ and IL-4 secreting CBMCs was also compared. The frequency of IFN-γ secreting CBMCs was significantly lower in helminth positive than negative participants to ESAT-6/CFP-10 cocktail (*p* = 0.018) but not to PPD (*p* = 0.37) *in vitro* stimulation (Figure 2A and 2B) respectively.

On the other hand, the median value of the frequency of IL-4 secreting CBMCs between helminth negative and positive remained insignificant to ESAT-6/CFP-10 (p = 0.64) and PPD (*p* = 0.38) stimulation respectively.

**Figure 3:**
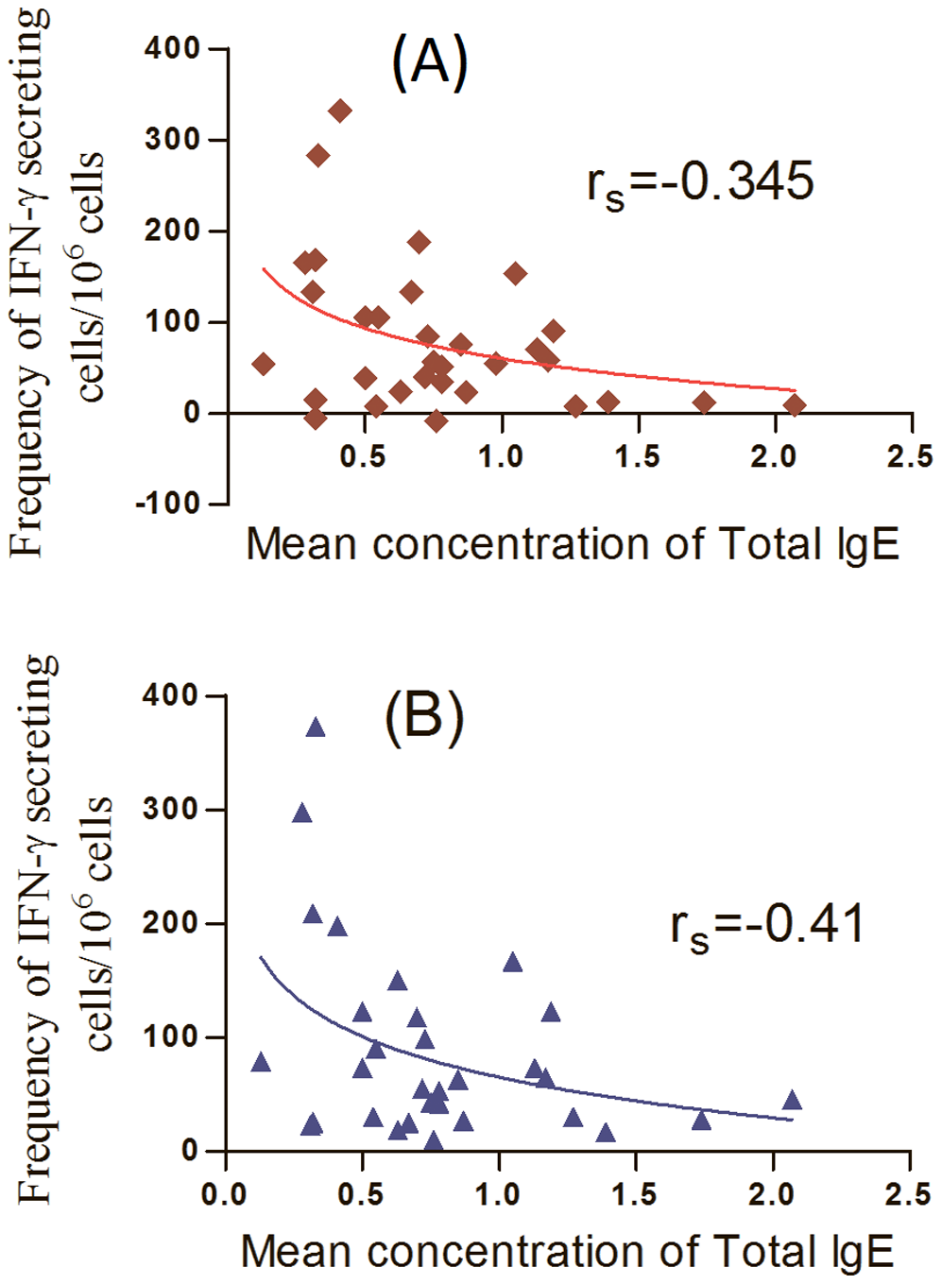
The relationship between the frequencies of IFN-γ secreting CBMCs (n = 40) in response to ESAT-6/CFP-10 cocktail (A) and PPD (B) with the concentration of total IgE of cord blood plasma (n = 40).

The relationship between IFN-γ secreting CBMCs to ESAT-6/CFP-10 cocktail and PPD stimulation with total IgE of the cord blood was also investigated using Spearman Rank Order Correlation coefficient separately. There was modest, negative correlation between the frequency of IFN-γ secreting cells in response to ESAT-6/CFP-10 cocktail (r_s_ = −0.35) and PPD (r_s_ = −0.41) stimulation and concentration of total IgE of cord blood between helminth positive and negative study participants (Figure 3A and 3B) respectively.

## Discussion

Protective immunity to TB is mainly cell mediated in which T_H_1 cells play central role [38]. Nevertheless, helminth infections can divert this T_H_1 type response through different mechanisms. For example, cross reactive helminth antigens may stimulate natural regulatory T (Treg) cells which down regulate the host immune response; and with increasing load of helminth antigens, a stronger regulatory network might be established through antigen-specific (adaptive) Treg cells [23], [24]. Therefore, chronic helminth infection might be one of the risk factors for increasing the susceptibility of the host to TB disease.

In this study, the prevalence of parasitic infections was relatively low (27.1% for helminth and 8.3% for protozoan) compared to previous studies conduct on school children in Mekelle city, northern part of Ethiopia where they showed 91% of helminth infection, (Ye'ebiyo Y *et al*, unpublished data) and in the deworming program by Wolday D *et al* [39]. This might be due to the difference in study participants (primary schools children) and collection site (sample was collected from children who had frequent contact with stagnant river water and ponds).

The prevalence of LTBI using QFT-GIT was 26.8% in our study. This result is similar to a study conducted in Tanzania (26.2%) though the method they used was Tuberculin Skin Test (TST) [3]. A community based LTBI prevalence study in the Afar region, Ethiopia was higher (63.7%) [40] than our finding. Other studies in the same site reported 59.3% among TB suspects using QFT-GIT assay [41]. A study conducted at Addis Ababa University on male medical students also showed that 43.9% was QFT-GIT positive which is higher than our result [42]. One possible explanation for this might be the immuno-suppressive effect of pregnancy [43]. Generally human newborns are deficient in their ability to produce protective humoral and cellular immune responses. This deficiency is thought to reflect physiologic immaturity of T and B cell functions and lack of previous exposure to exogenous antigens [44]. Although there is a view that cord blood IgE represents de novo neonatal IgE production, one cannot rule out the possibility of cross placental maternal source. We hypothesized that prenatal exposures to maternal helminth derived antigens elevate the level of total IgE in cord blood. Our result was consistent with the hypothesis; mean concentration value of total IgE was significantly higher in cord blood from helminth positive (0.76±0.59) than negative (0.46±0.43) study participants, *p* = 0.042. Ninety one percent of cord blood samples which had total IgE below the detection limit were found from helminth negative participants. This is supported by different studies conducted in helminth endemic areas in Africa [19], [33], [44]. Cord blood from 30% of Kenyan mothers had a detectable amount of total IgE whereas undetectable amounts were observed in all USA and Europeans cord blood samples [19], [28]. Increased amounts of cord blood total IgE from helminth positive mothers were also reported in many other studies [28], [33], [44], [45].

Though the major immunity against TB is the cell mediated arm of the immune system, the humoral immune response is also gaining increasing attention. IgG is the primary immunoglobulin which crosses the placenta [24]. In this study, we observed that helminth infection increased the cross placental transfer of TB specific IgG in LTBI positive study participants. Mean concentration of cross placental transfer of TB specific IgG, in cord blood plasma, was also compared between helminth negative and positive LTBI positive study participants. Our result showed nearly a 2-fold increase in the mean value of cord blood TB specific IgG between helminth positive and helminth negative LTBI positive study participants. The reason for this could be either an increase in plasma level or increased permeability of the placenta; this however requires further studies.

In this study, the median frequency of IFN-γ (80SFC/million) and IL-4 (10 SFC/million) secreting CBMCs in response to ESAT-6/CFP-10 cocktail antigens were slightly lower than to PPD stimulation (IFN-γ (88 SFU/million) and IL-4 (15 SFU/million)). This might be because PPD has less specificity than ESAT-6/CFP-10, as PPD is also found in other non-tuberculosis *Mycobacterium*. In majority of our study participants, IL-4 ELISpot indicated strong background response. This might be due to presence of other environmental antigens that induces T_H_2 response and stimulate the fetal immune system to produce more IL-4. Similarly, Demissie A *et al* had also observed that IL-4 background response was higher in Ethiopians (disease endemic country) than Danish participants [46]. The frequency of IFN-γ secreting CBMCs in response to ESAT-6/CFP-10 cocktail (p = 0.018) and PPD (*p* = 0.022) was significantly lower in helminth positive than helminth negative participants. Different studies reported similar findings with our study [17], [33], [47].

A number of studies indicated that maternal helminth infection associated with reduced IFN-γ response to *in vitro* stimulation with Mycobacterial antigens [17], [33], [47]. However different speculation was given on how this happened. Earlier studies reported that the reason for reduced IFN-γ response during chronic helminth infection was due to a shift in T_H_1/T_H_2 response, i.e. chronic helminth infection creates an environment that favored naïve T cells to differentiate and mature towards T_H_2 subsets [12], [48]. However other groups reported that though chronic helminth infection reduces IFN-γ response, the mechanism on how this happened was explained by increased Treg cell activity (increased secretion of suppressor cytokines, IL-10 and TGF-β) which is responsible for antigen specific or non-antigen specific hypo-responsiveness observed during chronic helminth infection [12].

In this study we did not observe statistically significant difference in the frequency of IL-4 secreting CBMCs between helminth positive and negative study participants to ESAT-6/CFP-10 cocktail (*p* = 0.64) and PPD (*p* = 0.38) upon *in vitro* stimulation. This might be related to environmental/host factors such as the presence of IL-4 inducing allergens. It is well recognized that a fine difference in IL-4 is difficult to establish because IL-4 is secreted such in a small quantities and/or consumed rapidly that accurate measurement is technically problematic [46]. In contrast to our result, in mouse model studies, it was reported that cytokines secreted by T_H_2 cells were profoundly increased while T_H_1cytokines reduced and authors concluded that this was due to T_H_1/T_H_2 shift [30], [34]. Later, by the same authors, disproved that reduction in T_H_1 cytokines response during chronic helminth infection might not be only due to T_H_1/T_H_2 shift but due to increased secretion of Treg cell cytokines (IL-10 and TGF-β) [12], [49], [50]. Another study also indicated that reduction in T_H_1 cytokines response during helminth infection was not associated with elevated level of IL-4 but due to increased level of IL-10 and TGF-β and this can be regained by treating with anti-TGF-β and anti-IL-10 antibodies [51].

Therefore, in our study, the frequency of IL-4 secreting CBMCs was more or less in agreement with other studies [49]–[52]. However, our study did not investigate as to whether the reduction on the frequency of IFN-γ secreting CBMCs from helminth positive study participants were due to increased activities of Treg cells or due to the T_H_1/T_H_2 shift. Moreover, deeper analysis based on each helminth species could not be made due to small sample size.

## Conclusion

The study showed an association between maternal helminth infections and increased total IgE and TB specific IgG of cord blood in helminth positive than negative study participants.

A number of studies, including ours, confirmed that chronic maternal helminth infection attenuated CBMCs to secret IFN-γ in response to *M. tuberculosis* antigens. On the other hand, data regarding IL-4 response of CBMCs from helminth infected subjects remains conflicting. Studies including ours showed that maternal helminth infection did not significantly associate with increased frequency of IL-4 secreting CBMCs to *M. tuberculosis* antigens. Therefore, the reason for the reduced IFN-γ response might not be due to T_H_1/T_H_2 shift but due to increased activity of regulatory T cells (IL-10 and TGF-β) as confirmed by other studies [30], [49], [50]. This needs further study to confirm the mechanism for reduced IFN-γ production by CBMC. We hope to further explore the impact of these findings as well as explore underlying mechanisms in a neonatal BCG vaccination model among pregnant women with variable exposure to helminth and *M tuberculosis* infections. Understanding this phenomenon would provide information on how best infant vaccination strategy should be designed.
